# Gut Microbiome and Metabolome Modulation by Maternal High-Fat Diet and Thermogenic Challenge

**DOI:** 10.3390/ijms23179658

**Published:** 2022-08-25

**Authors:** Henry A. Paz, Anna-Claire Pilkington, Ying Zhong, Sree V. Chintapalli, James Sikes, Renny S. Lan, Kartik Shankar, Umesh D. Wankhade

**Affiliations:** 1 Department of Pediatrics, College of Medicine, University of Arkansas for Medical Sciences, Little Rock, AR 72205, USA; 2 Arkansas Children’s Nutrition Center, Little Rock, AR 72202, USA; 3 Department of Pediatrics, Section of Nutrition, University of Colorado School of Medicine, Anschutz Medical Campus, Aurora, CO 80045, USA

**Keywords:** gut microbiota, thermogenesis, maternal high fat diet, CL316,243, cold exposure, metabolome

## Abstract

The gut microbiota plays a critical role in energy homeostasis and its dysbiosis is associated with obesity. Maternal high-fat diet (HFD) and β-adrenergic stimuli alter the gut microbiota independently; however, their collective regulation is not clear. To investigate the combined effect of these factors on offspring microbiota, 20-week-old offspring from control diet (17% fat)- or HFD (45% fat)-fed dams received an injection of either vehicle or β3-adrenergic agonist CL316,243 (CL) for 7 days and then cecal contents were collected for bacterial community profiling. In a follow-up study, a separate group of mice were exposed to either 8 °C or 30 °C temperature for 7 days and blood serum and cecal contents were used for metabolome profiling. Both maternal diet and CL modulated the gut bacterial community structure and predicted functional profiles. Particularly, maternal HFD and CL increased the Firmicutes/Bacteroidetes ratio. In mice exposed to different temperatures, the metabolome profiles clustered by treatment in both the cecum and serum. Identified metabolites were enriched in sphingolipid and amino acid metabolism in the cecum and in lipid and energy metabolism in the serum. In summary, maternal HFD altered offspring’s response to CL and altered microbial composition and function. An independent experiment supported the effect of thermogenic challenge on the bacterial function through metabolome change.

## 1. Introduction

A diverse microbiome plays a vital role in health and longevity. Key metabolic functions are aided by the microbiota, including—but not limited to—digestion, synthesis of essential nutrients, and regulation of energy balance [1,2]. Disruption of the gut microbial equilibrium is associated with the progression of metabolic abnormalities such as obesity and concurrent co-morbidities [3]. Microbial colonization of the gut primarily begins right after birth and is modulated by genetic and environmental factors, especially composition of the diet [4,5]. The intrauterine environment has a pivotal role in conferring disease risk in later life. Using animal models of diet-induced maternal obesity, we have shown that offspring of obese dams are hypersensitive to weight gain and exhibit insulin resistance, metabolic dysfunction, and hepatic steatosis when challenged with a high-fat diet (HFD) post-weaning [6,7]. In addition, we have also demonstrated the sexual dimorphic nature of programming of microbiome in offspring, suggesting the microbiome as a potential mechanism that contributes to developmental programming of fatty liver disease [8].

A large body of literature suggests that there are critical windows of development (preconception, early gestation, late gestation) where maternal obesity can program offspring organ development and physiology [9,10]. Adipose tissue is one such organ whose development hinges on maternal environment and can be programmed based on dietary/environmental challenges. Being a primary driver of metabolic dysfunction in the obese state, it is critical to understand the development and function of adipose tissue [11]. White adipose tissue (WAT) is known to store excessive energy in the form of lipids; whereas brown adipose tissue (BAT) is known for its thermogenic properties, which can potentially be used to counteract excessive weight gain and obesity. Increased fat mass and overall weight gain in offspring of obese dams are hallmarks of the metabolic programming phenomena [6,12].

Findings that linked impaired thermogenesis with obesity in murine models have led to an interest in brown fat as a candidate to counteract obesity [13]. However, in most humans, presence of brown fat—especially in the absence of stimulant such as cold—is debated. In rodents, cold exposure (typically between 4 and 18 °C) and chemical stimulation via β-adrenergic agonists such as CL316,243 (CL) are a couple of known ways to activate BAT and promote the appearance of brown-like/beige adipocytes in WAT, i.e., browning. Recent reports show that the gut microbial profile changes during cold exposure and microbiota transplantation from cold challenged mice increased WAT browning, energy expenditure, and tolerance to cold temperatures [14]. In microbiota-depleted (ABX) and germ-free mice, the thermogenic capacity of BAT has been observed to be impaired through decreased expression of uncoupling protein 1 (UCP1) and reduced browning in WAT [15]. Although these seminal studies have set the field of adipose tissue and microbiome on a proper course, the links between them require further elucidation.

To date, there are no studies exploring the interaction between maternal HFD and β3-adrenergic agonists such as CL on offspring gut microbiome composition. Generational effect of HFD on programming of microbiome and its possible interaction with CL warrants further research. Effects of other β3-adrenergic stimulants, such as cold temperature on microbial-derived metabolites, are also not thoroughly studied. Thus, the aim of this study was two-fold: First, to evaluate the effects on the gut microbiota composition in response to a combination of maternal HFD and CL. Second, using cold temperature as a β3-adrenergic stimulant, we wanted to assess its effect on microbiome and metabolome composition. We showed that both maternal HFD and CL challenge modulated the bacterial community structure and predicted functional profiles. We also demonstrated that thermogenic stimulus through cold exposure alone altered the cecum and serum metabolomes in mice. Taken together, these results suggest that maternal HFD could regulate thermogenic responses in offspring through perturbations on the gut bacterial community that promote changes in microbial-derived metabolites.

## 2. Results

### 2.1. Gut Microbial Diversity

**Figure 1:**
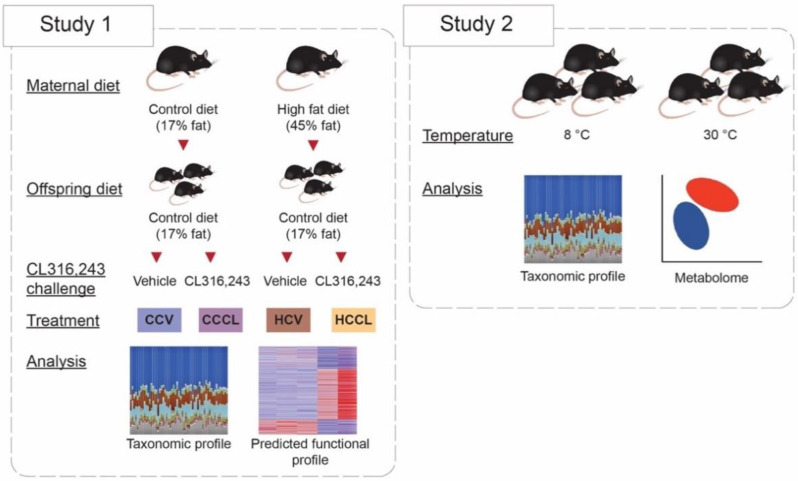
Overview of the conducted studies. In Study 1, CCV = offspring from control diet-fed dams treated with vehicle (n = 5), CCCL = offspring from control diet-fed dams treated with CL316,243 (n = 5), HCV = offspring from high fat diet-fed dams treated with vehicle (n = 4), HCCL = offspring from high fat diet-fed dams treated with CL316,243 (n = 4). In Study 2, mice were exposed to either 8 °C (n = 5) or 30 °C (n = 5) for 7 days.

Two studies were conducted to evaluate the effects of maternal diet and CL on offspring’s cecal bacterial community structure and predicted functional profile and to evaluate cold exposure effects on the cecal and serum metabolome profiles (Figure 1). α-diversity describes the structure of a microbial community through metrics of richness (number of taxa), evenness (relative abundance of the taxa), or integration of those two. In study 1, both the bacterial community richness and evenness were not influenced by maternal diet (*p* ≥ 0.14) or CL challenge (*p* ≥ 0.08) at the phylum- or genus-levels (Table 1). The Shannon diversity index marginally increased (*p* = 0.04) in offspring receiving CL compared to vehicle at the genus-level (2.73 ± 0.05 vs. 2.59 ± 0.05), but no changes were observed at the phylum-level by either maternal diet or CL challenge (*p* ≥ 0.15) (Table 1).

**Figure 2:**
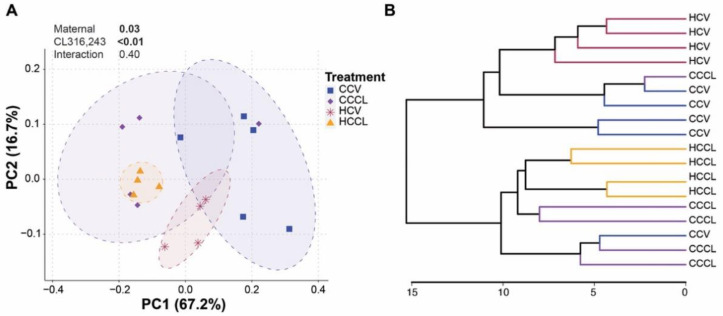
Murine gut bacterial community composition driven by maternal diet and CL316,243 challenge. (**A**) Genus-level principal coordinate analysis plot based on Bray–Curtis dissimilarities. Axes show the first two principal components and their corresponding percentage of variance explained. Ellipses define the 95% confidence level. Two-way PERMANOVA was used to determine the main effects of maternal diet, CL316,243 challenge and their interaction (statistically significant *p*-values are bolded). (**B**) Hierarchical dendrogram based on Ward’s method displaying clustering by treatment. CCV = offspring of control diet-fed dams treated with vehicle (n = 5); CCCL = offspring of control diet-fed dams treated with CL316,243 (n = 5); HCV = offspring of high-fat diet-fed dams treated with vehicle (n = 4); HCCL = offspring of high-fat diet-fed dams treated with CL316,243 (n = 4).

β-diversity describes the (dis-)similarity among microbial communities. Visualization of Bray-Curtis dissimilarities using the PCoA plot (Figure 2A) revealed clustering of bacterial communities by treatment with samples from offspring of HFD-fed dams (HCV and HCCL) displaying less variation compared to those from offspring of control diet-fed dams (CCV and CCCL). Based on PERMANOVA, maternal HFD and CL challenge significantly (*p* ≤ 0.03) impacted the bacterial community composition. To further assess bacterial community differences, a hierarchical clustering analysis was performed and the constructed dendrogram supported grouping based on maternal diet and postnatal CL treatment (Figure 2B).

### 2.2. Taxonomic Profile Differences

**Figure 3:**
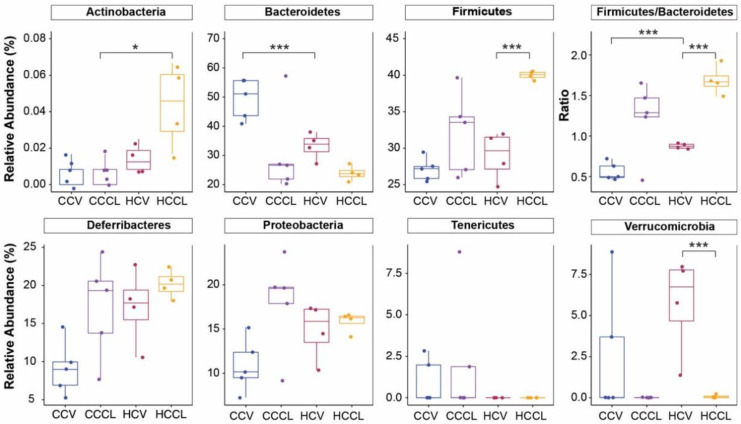
Phylum-level differences across treatments. CCV = offspring of control diet-fed dams treated with vehicle (n = 5); CCCL = offspring of control diet-fed dams treated with CL316,243 (n = 5); HCV = offspring of high-fat diet-fed dams treated with vehicle (n = 4); HCCL = offspring of high-fat diet-fed dams treated with CL316,243 (n = 4). Nonparametric multiple comparisons (* *p* < 0.05, *** *p* < 0.001).

Taxonomic analysis uncovered significant differences in the bacterial community composition driven by maternal diet or CL challenge (Figure 3). At the phylum-level, a maternal diet effect was observed in the mean relative abundance of Actinobacteria, Bacteroidetes, and the Firmicutes/Bacteroidetes ratio. For the vehicle-treated group, offspring of HFD-fed dams had a lower (*p* < 0.001) abundance of Bacteroidetes (33.2 vs. 49.4%) which resulted in a greater (*p* < 0.001) Firmicutes/Bacteroidetes ratio (0.88 vs. 0.56) compared to those of control-fed dams. For CL treated, offspring of HFD-fed dams had a greater (*p* ≤ 0.05) abundance of Actinobacteria (0.04 vs. 0.01%) compared to offspring of control-fed dams. The relative abundance of Actinobacteria in the cecum of C57BL/6 mice has been previously reported to be ~0.1% [16]. The CL challenge promoted an increase (*p* < 0.001) in the mean relative abundance of Firmicutes (40.0 vs. 29.0%) and the Firmicutes/Bacteroidetes ratio (1.69 vs. 0.88) and a decrease (*p* < 0.001) in the abundance of Verrucomicrobia (0.08 vs. 5.71%) in offspring of HFD-fed dams, whereas CL had no effect among phyla in offspring of control-fed dams.

**Figure 4:**
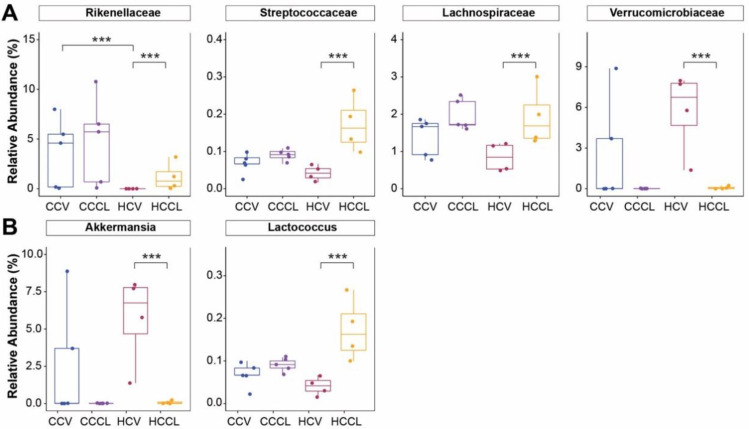
Family- and genus-level differences across treatments. Relative abundance of specific (**A**) families and (**B**) genera. CCV = offspring of control diet-fed dams treated with vehicle (n = 5); CCCL = offspring of control diet-fed dams treated with CL316,243 (n = 5); HCV = offspring of high-fat diet-fed dams treated with vehicle (n = 4); HCCL = offspring of high-fat diet-fed dams treated with CL316,243 (n = 4). Nonparametric multiple comparisons (*** *p* < 0.001).

At the family-level (Figure 4A), a maternal diet effect was observed for Rikenellaceae which had a greater (*p* < 0.001) mean relative abundance in offspring from maternal control diet compared to those from maternal HFD when treated with vehicle (3.66 vs. 0.0%). In offspring of maternal HFD, the CL challenge increased (*p* < 0.001) the abundance of Streptococcaceae (0.17 vs. 0.04%) and Lachnospiraceae (1.91 vs. 0.85%) and decreased (*p* < 0.001) the abundance of Verrucomicrobiaceae (0.08 vs. 5.71%) compared to vehicle. These responses were in line with those observed at the phylum-level. At the genus-level (Figure 4B), the abundance of Akkermansia decreased (*p* < 0.001; 5.71 vs. 0.08%), whereas the abundance of Lactococcus increased (*p* < 0.001; 0.04 vs. 0.17%) in response to the CL challenge in offspring of maternal HFD.

### 2.3. Predicted Functional Profiles

**Figure 5:**
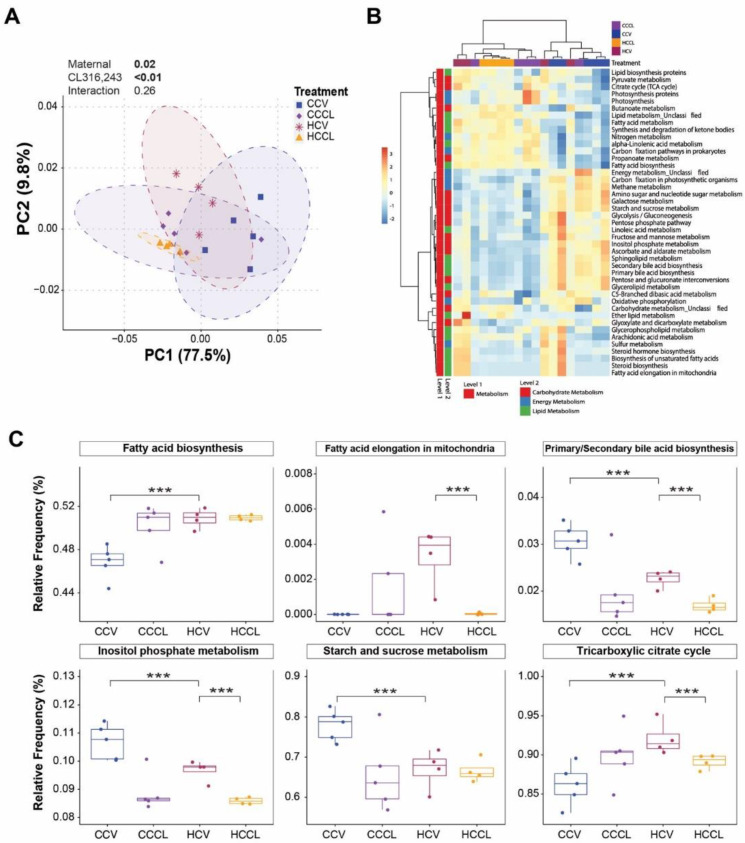
Distinct PICRUSt-predicted functional profiles in the mice gut driven by maternal diet and CL316,243 challenge. (**A**) Principal coordinate analysis plot based on Bray-Curtis dissimilarities generated from the pathways relative abundance. Axes show the first two principal components and their corresponding percentage of variance explained. Ellipses define the 95% confidence level. (**B**) Heatmap of pathways related to carbohydrate, energy, and lipid metabolism. (**C**) Significantly different metabolic-related pathways across treatments. Statistically significant *p*-values are bolded. CCV = offspring from control diet-fed dams treated with vehicle (n = 5); CCCL = offspring from control diet-fed dams treated with CL316,243 (n = 5); HCV = offspring from high fat diet-fed dams treated with vehicle (n = 4); HCCL = offspring from high fat diet-fed dams treated with CL316,243 (n = 4). Nonparametric multiple comparisons (*** *p* < 0.001).

Consistent with bacterial community differences, the predicted bacterial metabolic profiles clustered together per treatment as shown in the PCoA plot with the effects of maternal diet and CL challenge being significant (*p* ≤ 0.02; Figure 5A). The maternal diet effect on the metabolic profiles was more pronounced in offspring receiving vehicle compared to CL. Pathways associated with carbohydrate, energy, and lipid metabolism demonstrated hierarchical clustering by treatment (Figure 5B). To further evaluate these differences, the effects of maternal diet and CL challenge were determined on the metabolic profile of mice (Figure 5C). For the vehicle-treated group, metabolism-related pathways—such as fatty acid biosynthesis and tricarboxylic citrate cycle—increased (*p* < 0.001) while primary/secondary bile acid biosynthesis, inositol phosphate metabolism, and starch and sucrose metabolism decreased (*p* < 0.001) in offspring of HFD-fed dams compared to those of control diet-fed dams. For offspring of maternal HFD, the CL challenge decreased (*p* < 0.001) fatty acid elongation in mitochondria, primary/secondary bile acid biosynthesis, inositol phosphate metabolism, and the tricarboxylic citrate cycle.

### 2.4. Gut Microbial Diversity and Taxonomy from Mice Exposed to Different Temperatures

The number of taxa and their relative abundance at the phylum- and genus-levels were similar between mice exposed to 8 °C or 30 °C (Table 2). Mice exposed to cold temperature had a lower (*p* = 0.04) Shannon diversity at the phylum-level compared to mice exposed to thermoneutral temperature, but this effect was not consistent at the genus level (*p* = 0.74; Table 2).

**Figure 6:**
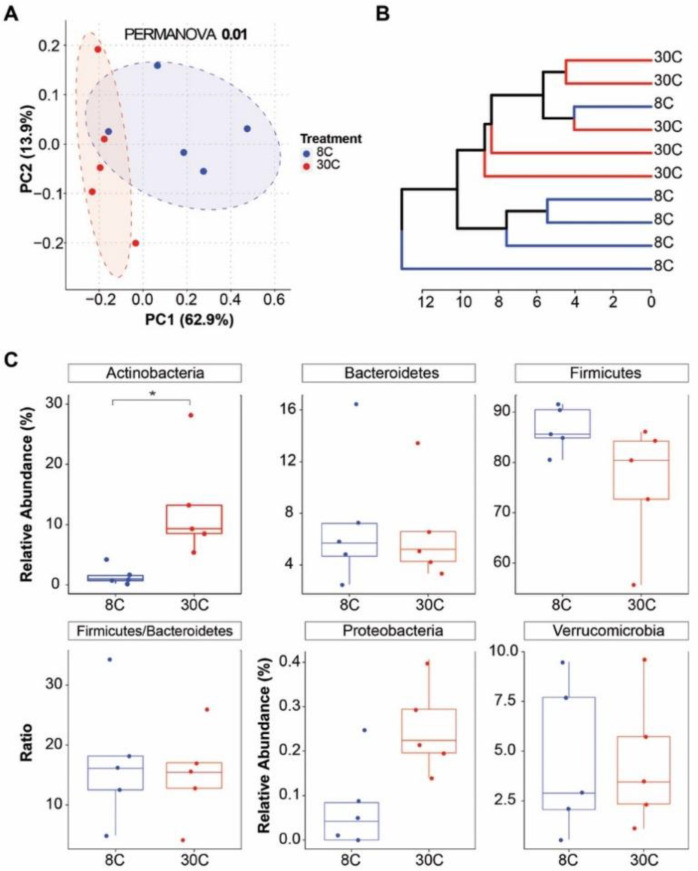
Beta diversity and phylum-level differences from the cecal bacterial community of mice exposed to different temperatures. (**A**) Genus-level principal coordinate analysis plot based on Bray-Curtis dissimilarities. Axes show the first two principal components and their corresponding percentage of variance explained. Ellipses define the 95% confidence level. (**B**) Hierarchical dendrogram based on Ward’s method displaying clustering by temperature. (**C**) Phylum-level classification of the cecal bacterial community. Mice exposed to either 8 °C (n = 5) or 30 °C (n = 5) for 7 days. Wilcoxon rank sum test (* *p* < 0.05).

The cecal bacterial community composition differed (*p* = 0.01) between mice exposed to cold and thermoneutral temperatures (Figure 6). This was clearly reflected through the clustering of samples by temperature in both the PCoA plot and dendrogram (Figure 6A,B). At the phylum-level, the relative abundance of Actinobacteria was lower (*p* < 0.05) in mice exposed to cold compared to thermoneutral temperature (1.53 vs. 12.9%; Figure 6C). The remaining phyla and the Firmicutes/Bacteroidetes ratio were similar between temperatures (Figure 6C).

### 2.5. Cecum and Serum Metabolomic Profiling

PICRUSt results provided a glimpse into the functional potential of the microbiome in offspring treated with CL. Unfortunately, we did not have access to either cecal content or serum from these mice to perform metabolomics. Thus, to understand how thermogenic challenge (β-adrenergic stimulation) would impact fecal and serum metabolomes, we performed an independent study where we exposed male mice to cold (8 °C, CE) and thermoneutral (30 °C, TN) temperatures. Then, untargeted metabolomics was performed on the cecal and serum samples from these mice.

**Figure 7:**
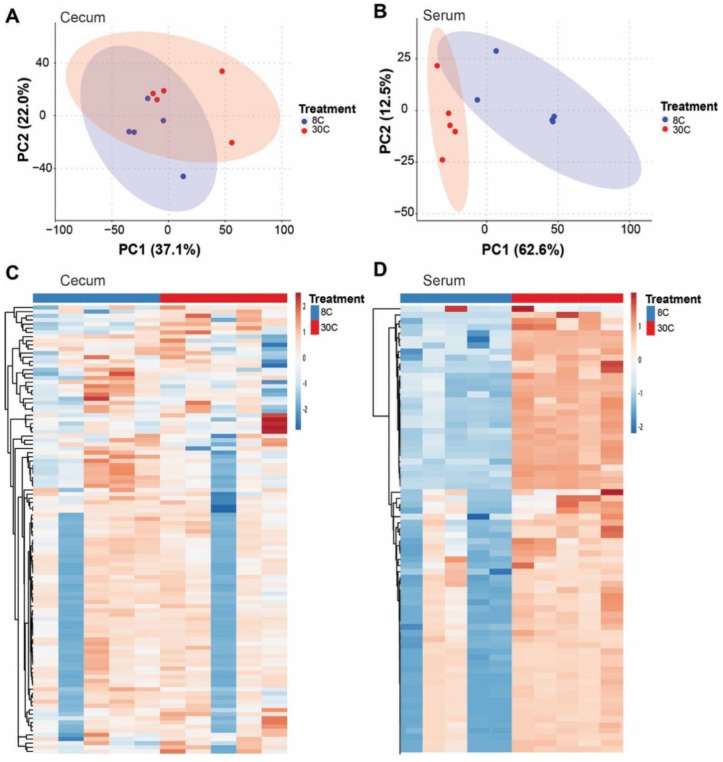
Metabolome profiles of the cecum and serum in mice exposed to different temperatures. Principal coordinate analysis plot based on Bray-Curtis dissimilarities including all metabolite features and showing clustering by temperature in the (**A**) cecum (n = 5) and (**B**) serum (n = 5). Ellipses define the 95% confidence level. Heatmaps from identified metabolite features in the (**C**) cecum and (**D**) serum.

**Figure 8:**
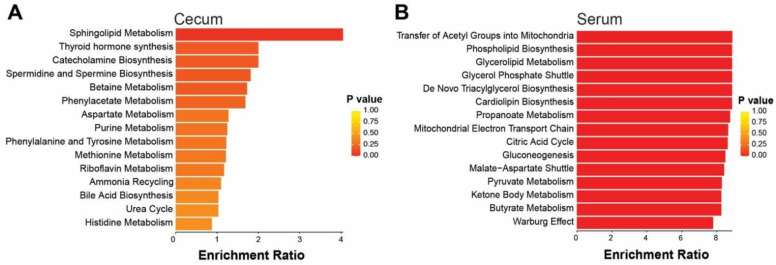
Metabolic pathways (top 15) in the (**A**) cecum and (**B**) serum associated with enriched metabolites in mice exposed to cold (8 °C) or thermoneutral (30 °C) temperatures. Quantitative enrichment analysis was performed on identified metabolites in the cecum (n = 108) and serum (n = 75) using MetaboAnalyst v5.0.

The PCA of all the detected metabolite features (known/unknown) showed clustering of samples by temperature (Figure 7A,B). Heatmaps of identified metabolites revealed more pronounced differences in the serum (75 identified metabolites) compared to the cecum (108 identified metabolites) (Figure 7C,D). The quantitative enrichment analysis was performed on known metabolites to identify enriched functionally related metabolites and their associated pathways. In the cecum, the relative abundance of D-sphingosine was greater (*p* = 0.05) in CE mice compared to TN mice (Figure S2A); while in the serum, the relative abundance of citric acid, glycerol 3-phosphate, fumaric acid, succinic acid, isocitric acid, β-alananine, and lactic acid were lower (*p* < 0.001) in CE mice compared to TN mice (Figure S2B–H). In the cecum, sphingolipid metabolism pathways involved in specific amino acids metabolism and bile acid biosynthesis were among the top 15 pathways associated with enriched metabolites (Figure 8). Bile acid biosynthesis was highlighted in the bacterial predicted pathways from the CL treated offspring of HFD-fed dams (Figure 5C) which is consistent with findings from the CE mice metabolome in the second experiment. Although we agree that this is not an exact replication of the experiment due to the lack of the maternal factor. The systemic nature of these changes was evident in that more pathways were significantly affected in the serum compared to the cecum (Figure 8).

## 3. Discussion

Maternal programing and its role in shaping metabolic dysfunction is well demonstrated [17]. Alterations in the gut microbiome are linked to metabolic dysfunctions that are present in obesity [18,19]. Mounting evidence supports an effect of maternal HFD on the offspring’s gut microbiota composition and consequently on important metabolic organs such as liver [8,20]. Although recent studies have established a connection between dysbiosis of gut microbiota and adipose tissue function [14,21], the effect of β-adrenergic challenges such as CL on the microbiome composition and its interaction with maternal HFD is unknown. Using a mouse model of maternal diet-induced obesity, we demonstrated that maternal HFD and CL challenge affected the microbiome composition in offspring. We also observed that cold exposure promoted changes in the metabolome profile that were more pronounced in the serum compared to the cecum. This study provides insight into how maternal programming shapes the microbiome and impacts postnatal response to CL challenges and shows the ability of ambient temperature to change the metabolome in mice.

In the current study, maternal HFD promoted marked differences in β- but not α-diversity in the offspring’s cecal microbiota, which is consistent with previously reported results from ours and other groups [7,22,23]. Similarly, findings from several human studies revealed that there are differences in the gut microbiota composition between infants from overweight and normal weight women suggesting a relationship between maternal dietary habits, gestational weight, and programming of offspring microbiome [24,25]. In children born to obese mothers, the gut microbiota has been observed to display taxonomic compositional changes, greater homogeneity, and species diversity compared to those born to non-obese mothers; although these responses can be impacted by the socioeconomic status of mothers [26,27]. Firmicutes and Bacteroidetes are the dominant phyla in the human gut [28]. The Firmicutes/Bacteroidetes ratio changes with age and has been commonly observed to increase with obesity; however, its use as a marker of gut dysbiosis is currently debated [29,30,31]. Furthermore, variations in microbial abundances may not always impact the functional capacity due to functional redundancy [32]. However, maternal high-fat diets have been shown to increase the Firmicutes/Bacteroidetes ratio in the mother with persistent effects observed in early stages in the offspring [22,33]. A negative correlation was reported between the relative abundance of Bacteroides species (within the Bacteroidetes phylum) in children and maternal fat intake during pregnancy [20]. Consistent with the aforementioned literature, in this study maternal HFD promoted decreased Bacteroidetes, increased Firmicutes, and consequently increased the Firmicutes/Bacteroidetes ratio in offspring.

Both CL challenge and cold exposure significantly affected β-diversity, but their impact on the taxonomic profile differed. CL and cold exposure are thermogenic stimulants that mediate their actions through β-3 adrenergic receptors with metabolic responses that can differ [34]. In addition, differences between conducted studies could be attributed to factors such as maternal diet background (maternal factor vs. no maternal factor), housing temperature (22 °C vs. 8 °C and 30 °C) and age (24-week-old mice and 16-week-old mice) [8,14,35]. However, the impact of β-adrenergic stimuli on the bacterial community structure was clear.

A healthy gut microbial community benefits the host’s metabolic homeostasis and reciprocally, the host can influence the microbial composition through gut environmental conditions, nutrients from the diet, chemicals, and gut hormone secretion among others [14,36,37,38]. In our study, we demonstrated that CL—a β3-adrenergic receptor agonist, known to activate BAT and to induce browning in WAT—impacts the gut microbiota composition. CL had significant effect on the relative abundance across taxonomic levels. Li et al. [15] showed that mice lacking gut microbiota had impaired thermogenesis in response to acute cold exposure as well as CL. These findings support the notion that the microbiota could contribute to the regulation of thermogenesis. In the current study, the CL challenge altered the relative abundance of taxonomic groups in offspring of maternal HFD. Similar shifts in the cecal microbiota such as an increase in Firmicutes and a decrease in Verrucomicrobia were reported in mice under thermogenic stimulus by cold exposure [14]. A decrease in Akkermansia muciniphila has been suggested to enable the adaptive increase in the intestinal surface during cold exposure [14]. We observed a near depletion of the genus Akkermansia in offspring exposed to the CL challenge. Worthmann et al. [39] has demonstrated that cold induced thermogenesis triggers a metabolic program that orchestrates lipoprotein processing in BAT and hepatic conversion of cholesterol to bile acids accompanied by distinct changes in gut microbiota. This is a plausible mechanism of thermogenesis-induced changes in the gut microbiota.

Bile acids secreted into the intestinal lumen in response to a meal intake are modified by the gut flora and act as messengers between gut microbiota and adipose tissue [40]. Additionally, bile acids themselves can regulate the composition of the gut microbiota [41]. Our group had previously identified significant correlations between bacterial families and bile acid species in the cecum of offspring of HFD-fed dams [8]. In line with the latter, in the current study changes in the predicted functional profile by the CL challenge involved significant differences in primary/secondary bile acid biosynthesis in offspring of HFD-fed dams. Furthermore, cold exposure led to changes in the cecal and serum metabolomes. Bile acid biosynthesis was among the pathways affected in the cecum metabolome which supported the predictive functional change from the gut microbiome by CL, which mimics cold exposure causing thermogenic stress.

Gut microbiome plays a critical role in composition of gut and serum metabolome through primary or secondary synthesis of metabolites such as bile acids and short chain fatty acids (SCFA) among others. SCFA levels are positively associated with increased energy metabolism [42,43,44], while other microbially derived metabolites—such as trimethylamine and imidazole propionate—show negative associations with host health [45,46,47]. In our study where we exposed mice to cold (8 °C) and thermoneutral (30 °C) temperatures, we showed differences in the metabolome profile that were more pronounced in the serum compared to the cecum. From the identified metabolites, pathways involved in sphingolipid and amino acid metabolism and bile acid biosynthesis were impacted by cold exposure in the cecum, whereas pathways involved in lipid and energy metabolism were impacted in the serum. During acute cold exposure (4 h), pathways related to amino acid metabolism and redox regulation have been shown to be impacted in BAT [48]. Indeed, under cold exposure, increased bile acid synthesis has been shown to alter the gut microbiota composition [39] and as observed in this study resulting in different cecal and serum metabolite profiles that could be linked to thermogenic responses. These findings were derived from the study where there was no maternal HFD impact. Activation of thermogenic adipose tissues is being considered as a potential opportunity to reduce obesity and metabolic disorders. β-adrenergic stimulation has been a target of therapeutic strategies due to its importance in the thermogenic response. In humans, β3-adrenergic agonists have the potential to promote thermogenesis and increase resting energy expenditure [49]; however, negative side effects have hindered their use in clinical trials [50]. Our current study also suggests that as β3-adrenergic agonists continue to develop, evaluation of their interaction with the gut microbiota is relevant as microbially derived metabolites can influence their response.

While the conducted studies showed that CL and cold exposure impact the cecal bacterial community structure, the studies presented some limitations. Direct comparison of the bacterial changes from β-adrenergic stimuli were not conducted due to the differences in experimental conditions between studies—such as maternal background, collection age, and housing temperatures. Metabolome analysis was only performed in mice exposed to different temperatures (Study 2) and not in mice of different maternal dietary background and receiving vehicle or CL (Study 1), thus it was not possible to contrast metabolome responses to CL and cold exposure. The untargeted metabolomics approach provides a semiquantitative analysis, therefore the absolute concentration differences between the significantly distinct metabolites are unknown. However, the objective was to determine whether β-adrenergic stimulus altered the metabolome profiles of the cecum and serum and not to compare metabolome responses between different β-adrenergic stimuli. Furthermore, metabolome results from this study provide insights of metabolites from the gut microbiota that could explain adipose tissue function during thermogenic stimuli. Previously, it has been shown that metabolic responses differed between CL and cold exposure in white adipose tissue [34], this poses a likely possibility of different metabolic response from the microbiome, metabolome, and other tissues. The current studies provide a primary evaluation of the effects of maternal diet and β-adrenergic stimuli on the gut microbial profile and the gut and serum metabolome profiles. Further research is needed to elucidate the links between microbiome and metabolome changes promoted by maternal dietary background or β-adrenergic stimuli and how these responses impact adipose tissue.

## 4. Materials and Methods

### 4.1. Experimental Design

The Institutional Animal Care and Use Committee at the University of Arkansas for Medical Sciences (IACUC #4094) approved all experimental protocols used in the two studies described in this manuscript.

#### 4.1.1. Study 1

Five-week-old female C57BL6/J mice (stock 0664, Jackson Laboratories, Bar Harbor, ME, USA) housed under conventional conditions were given ad libitum access to control diet (17% fat Harlan Teklad TD95095, n = 10) or HFD (45% fat, TD08811, n = 10) for 12 weeks. At 17 weeks of age, females were bred with lean male mice (fed control diet TD8640). This protocol was identical to those published previously by our group [7,8]. Upon birth, all offspring remained with birth dams until weaning and litter sizes were adjusted to 6 pups per litter. Female offspring were then separated and used in a different experiment and male offspring from control diet and HFD-fed dams were given access to the control diet which led to two groups of offspring: viz. offspring born to control diet-fed dams weaned onto control diet (CC, n = 10) and offspring born to HFD-fed dams weaned onto control diet (HC, n = 8). On week 20, mice received a daily intraperitoneal injection (1 mg/kg body weight) of either vehicle (CCV, n = 5; HCV, n = 4) or β3-adrenergic agonist CL (CCCL, n = 5; HCCL, n = 4) for 7 days (Figure 1). Food intake was not recorded on this study. On the morning of the day 8, mice were euthanized by carbon dioxide asphyxiation and cecal contents were collected and immediately snap frozen for further analysis.

#### 4.1.2. Study 2

Results from Study 1 provided a glimpse into the functional potential of the microbiome in offspring treated with CL. Unfortunately, we did not have access to either cecal content or serum from these mice to perform metabolomics. Thus, to understand the impact of thermogenic challenge (β-adrenergic stimulation) on cecal and serum metabolomes, 16-week-old male C57BL6/J mice (Jackson Laboratories, Bar Harbor, ME, USA) housed under conventional conditions were exposed to either 8 °C (cold exposure, n = 5) or 30 °C (thermoneutral, n = 5) temperature for 7 days (Figure 1). Mice were housed individually using environmental chambers (Power Scientific Inc., Doylestown, PA, USA). On the morning of the day 8, mice were euthanized and cecal contents were collected as described in Study 1. In addition, blood was collected via cardiac puncture and serum was separated and stored at −20 °C until further analysis.

### 4.2. Microbial Community Profiling Using 16S rRNA Amplicon Sequencing

Genomic DNA was extracted from the cecal samples using the MO BIO PowerSoil DNA Isolation kit (Qiagen, MD, USA, Catalog # 12955-4) with a few modifications. 7 Cecal contents (20–25 mg) were added directly onto 96-well plates with beads and recommended buffers in the wells. Sealed plates were shaken horizontally at 20 Hz for 20 min using a mixer mill (Retsch MM 400). The remaining steps were performed according to the manufacturer’s protocol. Extracted DNA was quantitated spectrophotometrically and stored at −20 °C. Fifty nanograms of genomic DNA were utilized for amplification of the V4 variable region of the 16S rRNA gene using the 515F/806R primers. Forward and reverse primers were dual-indexed as described by Kozich et al. [51]. Paired-end sequencing (2 × 250 bp) of pooled amplicons was carried out on an Illumina MiSeq with ~30% PhiX DNA.

### 4.3. Bioinformatics Analysis

Processing and quality filtering of reads were performed by using scripts in QIIME (v1.9.1) [52] and other in-house scripts [7]. Paired reads were stitched with PEAR, an over-lapping paired-end reads merger algorithm which evaluates all possible paired-end read overlaps minimizing false positive hits [53]. Reads were further filtered based on Phred quality scores and for chimeric reads using USEARCH61 [52,54] resulting in an average of 20,700 quality-filtered reads across samples and filtered reads were demultiplexed using QIIME. UCLUST was used to cluster sequences into operational taxonomical units (OTUs based on >97% identity) [54]. OTU picking was performed using an open-reference method which encompasses clustering of reads against a reference sequence collection and which also performs de novo OTU picking on the reads which fail to align to any known reference sequence in the database [55]. To eliminate erroneous mislabeling, the resulting OTU tables were checked for mislabeling sequences [56]. Overall, quality-filtered reads clustered into 238 OTUs across samples. Representative sequences were further aligned using PyNAST with the Greengenes core-set alignment template [57]. Construction of the phylogenetic tree was performed using the default (FASTTREE) method in QIIME [58]. Samples were rarefied to an even sampling depth of 12,000 quality-filtered reads and OTU richness was evaluated via rarefaction curves showing similar coverage across treatments (Figure S1) and sample community completeness was evaluated via Good’s coverage estimating that 99.8% of the bacterial diversity was characterized across samples. Alpha diversity metrics for richness, diversity, and evenness at the phylum-, genus-, and OTU-levels were determined in QIIME using the observed features, Shannon diversity index, and Pielou’s evenness index, respectively. Bray–Curtis dissimilarities were used to evaluate beta diversity and to conduct the principal coordinate analysis (PCoA). For predicted functional composition, OTUs were normalized by the predicted 16S copy number, and functions were determined using the GreenGenes 13_5 database for KEGG orthologs. The phylogenetic investigation of communities by reconstruction of unobserved states (PICRUSt) algorithm was used to identify differences in the predictive functional composition [59]. Relative frequencies from predicted pathways were visualized via a heatmap constructed using ClustVis [60].

### 4.4. Untargeted Metabolomics

Metabolomic sample preparation—Serum metabolome (100 µL) was extracted in cold methanol (400 µL). Instrumental pooled quality control (QC) samples were prepared by pooling equal volumes of each sample extract (20 µL). Samples and QC extracts were dried on a SpeedVac, reconstituted in 100 µL of 5% methanol spiked with a 1 ppm internal standard (Lorazepam; Sigma Aldrich, St. Louis, MO, USA) and subjected to LC-MS analysis. Cecal samples (75 mg) were dried overnight in a SpeedVac with an average dried cecal content of ~21 mg. Samples were homogenized in 600 µL 80% methanol using a Precellys 24 homogenizer (Bertin Corp.; Rockville, MD, USA) at 6500 rpm for two 30-s cycles. Samples were chilled on the dry ice for 8 min in between cycles. Experimental pools, samples, and QC extracts were then prepared as described for serum samples.

Chromatography—The Dionex Ultimate 3000 UHPLC was used with a XSelect CSH C18 reversed phase column (2.1 × 100 mm, 2.5 µm) kept at 49 °C, as previously described [61]. Metabolites were eluted by use of the following step gradient at a flow rate of 0.4 mL/min: 0–2 min, 0–1% solvent B; 2–6.5 min, 1–20% solvent B; 6.5–11.5 min, 20–95% solvent B; 11.5–13.5 min, 95–99% solvent B; 13.5–16.5 min, 99–100% solvent B; 16.5–20 min, 1% solvent B. Solvent A is 0.1% formic acid in water and solvent B is 0.1% formic acid in acetonitrile. A 5 µL of each sample extract was injected in a randomized sequence with one QC injection in every 5 samples. Metabololomic features were quantified using a Q Exactive Orbitrap mass spectrometer with both positive and negative electrospray ionization (ESI+/−) in full scan MS mode executed with Xcalibur 4.0 software. The ESI+/− data dependent MS2 spectra were acquired using QC samples for metabolite identification. The instrumentation detail was described previously [62].

Acquired data (full MS and data dependent MS2) were processed by Compound Discoverer 3.0 using an untargeted metabolomics workflow. In-depth details of parameters associated with this workflow have been published previously [62]. Metabolites were identified by using our in-house library containing MS1 spectra of 420 standard compounds with 5 ppm mass accuracy ± 15 s of retention time, mzVault (inhouse ddMS2 database), and mzCloud (online MS2 database) and given the following confidence levels: Level 1, identification if accurate mass, retention time, and MS2 spectra matching to our in-house (mzVault) library; and Level 2, identification if accurate mass, MS2 matching to known standard from our in-house library (score > 70) or mzCloud library (score > 80), and no retention time information.

### 4.5. Statistical Analyses

Statistical analyses were performed in R version 4.0.5 [63]. α-diversity metrics were evaluated for normality using the Shapiro–Wilk test and were analyzed using a two-way analysis of variance (ANOVA) to determine the main effects of maternal diet and CL challenge and their interaction or the unpaired *t*-test were appropriate. To evaluate differences in the relative abundance of taxonomic groups among treatments, specified nonparametric multiple comparisons using the “nparcomp” package [64] or the Wilcoxon rank sum test were conducted were appropriate. Differences in β-diversity were determined with Bray–Curtis dissimilarities and evaluated using the multivariate analysis of variance (PERMANOVA) with 999 permutations using the “vegan” package [65]. Bray–Curtis dissimilarities were inputted to construct a dendrogram using the Ward’s method for hierarchical clustering.

For metabolites detected in both ionization modes in serum and cecum, the ones with the greater intensity and lower %RSD were selected. Cecum data were normalized by sample weight and both cecum and serum data were log transformed (base 10) and auto scaled (mean-centered and divided by the standard deviation of each variable). To evaluate metabolome differences between temperatures, a principal component analysis was performed on all the data (identified and unidentified metabolites). Further quantitative enrichment analysis was performed only including the identified metabolites data in MetaboAnalyst v5.0 (https://www.metaboanalyst.ca/home.xhtml, accessed on 1 May 2022) using the Small Molecule Pathway Database (SMPDB) [66,67]. Statistical significance was determined at *p* < 0.05.

## 5. Conclusions

In summary, we showed that both HFD-induced maternal obesity and administration of the β3-adrenergic agonist CL altered the bacterial community and predicted functional profiles in offspring. Furthermore, offspring born to HFD fed dams were more responsive to bacterial profile changes compared to their counterparts when treated with CL. We also demonstrated that β-adrenergic stimulation via cold exposure alone can modulate the metabolomic profiles of the cecum and serum in male mice in the absence of maternal HFD feeding. Given that the gut microbiota plays an important role in the host metabolic homeostasis, changes in microbially derived metabolites can influence cross-talking with individual tissues such as WAT and BAT and can be considered as a proposed mechanism.

## Figures and Tables

**Table 1:** Alpha diversity indices at the phylum and genus levels.

Level	Index	Treatment ^1^				*p*-Value ^2^		
		CCV	CCCL	HCV	HCCL	Maternal	CL316,243	Interaction
Phylum	Observed OTUs	5.80 ± 0.37	6.00 ± 0.55	6.00 ± 0.01	5.75 ± 0.25	0.95	1.00	0.58
	Pielou’s evenness	0.72 ± 0.04	0.76 ± 0.03	0.81 ± 0.03	0.76 ± 0.02	0.14	0.92	0.14
	Shannon index	1.81 ± 0.09	1.95 ± 0.11	2.10 ± 0.06	1.92 ± 0.01	0.15	0.96	0.09
Genus	Observed OTUs	21.8 ± 0.97	23.0 ± 1.18	21.3 ± 0.85	23.5 ± 0.29	0.98	0.10	0.59
	Pielou’s evenness	0.57 ± 0.01	0.61 ± 0.02	0.60 ± 0.01	0.59 ± 0.01	0.48	0.17	0.06
	Shannon index	2.51 ± 0.07	2.76 ± 0.10	2.66 ± 0.04	2.70 ± 0.02	0.52	**0.04**	0.14

^1^ CCV = offspring of control diet-fed dams treated with vehicle (n = 5); CCCL = offspring of control diet-fed dams treated with CL316,243 (n = 5); HCV = offspring of high-fat diet-fed dams treated with vehicle (n = 4); HCCL = offspring of high-fat diet-fed dams treated with CL316,243 (n = 4). Data are expressed as mean ± SEM. ^2^ Two-way ANOVA was used to determine the main effects of maternal diet, CL316,243 challenge and their interaction (statistically significant *p*-values are bolded).

**Table 2:** Alpha diversity indices at the phylum and genus levels from the cecum of mice exposed to different temperatures.

		Treatment ^1^		
Level	Index	8 °C	30 °C	*p*-Value ^2^
Phylum	Observed OTUs	4.60 ± 0.24	5.00 ± 0.01	0.14
	Shannon index	0.71 ± 0.07	1.09 ± 0.14	**0.04**
	Pielou’s evenness	0.32 ± 0.03	0.47 ± 0.06	0.06
Genus	Observed OTUs	26.4 ± 0.51	26.6 ± 1.33	0.89
	Shannon index	2.70 ± 0.23	2.80 ± 0.17	0.74
	Pielou’s evenness	0.57 ± 0.05	0.59 ± 0.03	0.73

^1^ Mice exposed to either 8 °C (n = 5) or 30 °C (n = 5) for 7 days. Data are expressed as mean ± SEM. ^2^ Unpaired *t*-test (statistically significant *p*-values are bolded).

## Data Availability

Data available at https://doi.org/10.5281/zenodo.7020318.

## Supplementary Materials

The following supporting information can be downloaded at: https://www.mdpi.com/article/10.3390/ijms23179658/s1.
