# Supplementary material for: Gut Microbiome and Metabolome Modulation by Maternal High-Fat Diet and Thermogenic Challenge
Source: Int J Mol Sci. 2022 Aug 25;23(17):9658. doi: 10.3390/ijms23179658 (PMC9456050; doi:10.3390/ijms23179658)
Supplement: Supplementary file 1 [file ijms-23-09658-s001.zip › ijms-1784812-supplementary.pdf]

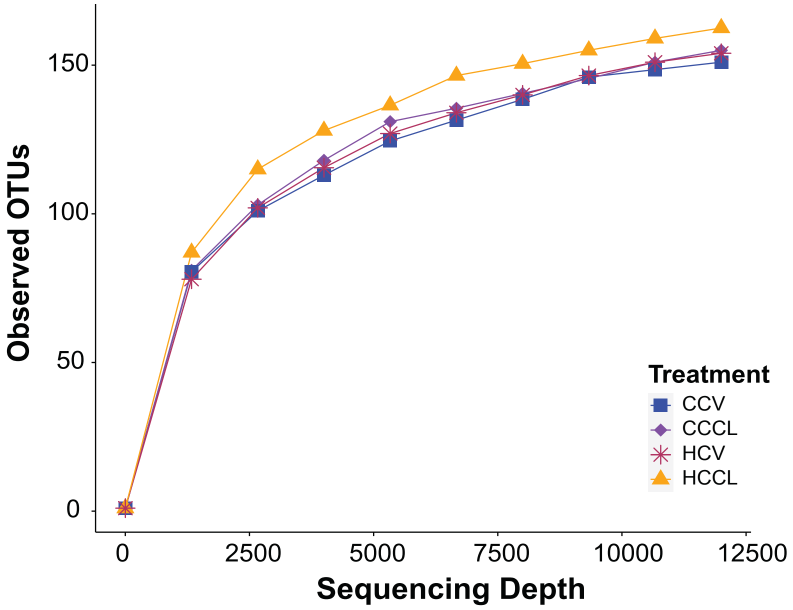

Figure S1. Rarefaction curves at an even sampling depth (12,000 quality-filtered reads) displaying similar bacterial community characterization across treatments. Each symbol represents the median value from 10 iterations. CCV = offspring of control diet-fed dams treated with vehicle (n = 5), CCCL = offspring of control diet-fed dams treated with CL316,243 (n = 5), HCV = offspring of high-fat diet-fed dams treated with vehicle (n = 4), HCCL = offspring of high-fat diet-fed dams treated with CL316,243 (n = 4).

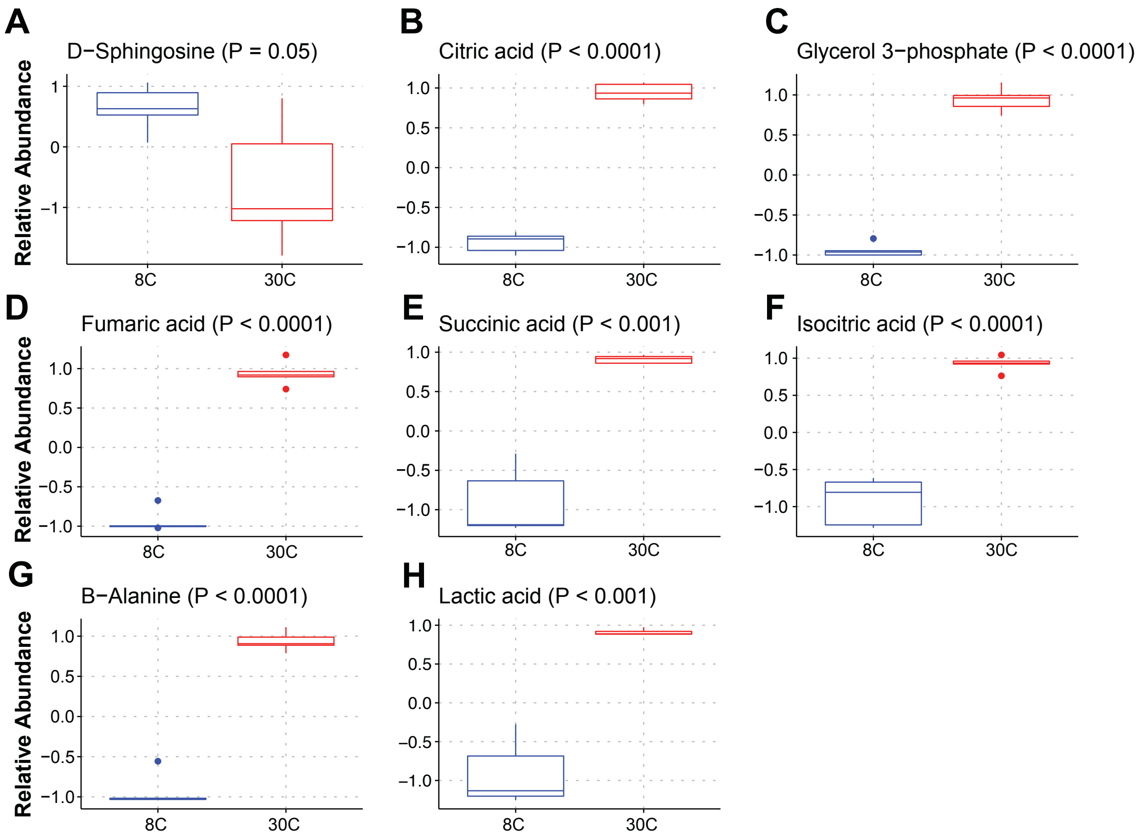

Figure S2. Distinct metabolite profile from identified metabolites in the (**A**) cecum and (**B-H**) serum from mice exposed to different temperatures. Student's unpaired, two-tailed t-test was used for temperature comparisons.
